# Factors Associated with the Nutritional Status among Male Workers of Iron and Steel Industries in Bara District, Nepal

**DOI:** 10.1155/2020/7432716

**Published:** 2020-06-12

**Authors:** Raj Kumar Sangroula, Hari Prasad Subedi, Kalpana Tiwari

**Affiliations:** College of Applied Food and Dairy Technology, New Baneshwar, Kathmandu, Nepal

## Abstract

**Background:**

Overweight and obesity are major serious public health problems, since their prevalence is accelerating rapidly not only in developed but also in developing countries. The aim of this study was to find out the factors associated with the nutritional status of the industrial workers in Bara District of Nepal.

**Methods:**

An industry-based analytical cross-sectional study was conducted among the 271 male workers using pretested semistructured questionnaires, food frequency questionnaire, 24-hour recall method, and anthropometric measurement after obtaining informed consent from the workers. For the categorical independent variables, bivariate and multivariate regression tests were used for the analysis, and for numerical independent variables, Student's *t*-test was used. A *P* value less than 0.05 was considered significant. Ethical approval was taken from the Research Committee of the College of Applied Food and Diary Technology (CAFODAT).

**Results:**

Overweight /obesity was observed among 27.3% of the participants of which 22.1% were overweight and 5.2% were obese. Age (OR: 2.54; 95% CI: 1.346–4.823); ethnicity, Brahmin/Chhetri (OR: 6.14; 95% CI: 1.971–19.123) and Madhesi (OR: 4.641; 95% CI: 1.534–14.04); and smoking (OR: 4.165; 95% CI: 1.972–8.80) were associated with nutritional status of industrial workers. Additionally, food frequency (OR: 2.232; 95% CI: 1.101–4.522), dietary diversity, and total calorie intake were also significantly associated with nutritional status of industrial workers.

**Conclusions:**

The study has indicated that more than one-fourth of workers of iron and steel industries in Bara District of Nepal are overweight or obese. Different sociodemographic and socioeconomic factors and lifestyle-related factors were associated with overweight and obesity. There is need for programs for industrial workers focused on nutrition education to raise awareness about nutrition-related problems and risk factors.

## 1. Introduction

Science and technology have made people's life easier, increased their life expectancies, and altered the lifestyle due to which different health problems like overweight and obesity arise [1–3]. Overnutrition has been a serious public health problem worldwide. Between 1975 and 2016, the worldwide prevalence of obesity has nearly tripled. According to the WHO, in 2016, more than one-third (39%) of adults of age 18 years or more were overweight and 13% were obese [4]. In a developing country like Nepal, the problem of obesity and overweight is rising [5]. According to the Nepal Demographic and Health Survey 2016, more than one-sixth (17%) of the males are overweight or obese. Of the total males, 15% are overweight and almost 3% are obese. The proportion of females who were overweight or obese was 9% in 2006 and increased to 13% in 2011 and 22% in 2016 [6]. A recent study conducted in 2019 by the Nepal Health Research Council has found that 23.5% of the adult population are overweight, 7.2% are obese, and 30.7% are either overweight or obese [7]. Overweight/obesity has independently been the risk factors for many noncommunicable diseases such as diabetes, hypertension, and cardiovascular diseases. [8, 9].

Industrial development has been the basis of sustainable economic development globally, and for this, the government of Nepal has also given emphasis on the industries since 1950 [10]. Many of the industries in Nepal are agroprocessing, and very few are producing construction materials and export-oriented products [10]. According to the Nepal Labour Force Survey 2017/2018, almost 7.1 million of Nepal population are employed and almost one-fifth population of them were employed in agriculture, the biggest employing industry followed by trade industry (17.5 percent), and construction (13.8 percent) [11]. The concept of occupational health and safety is still new in a developing country like Nepal which mainly concentrates on occupation-related diseases [10]. The Labour Act of Nepal, 2017, has given emphasis on working hours, occupational health and safety, and use of personal protective equipment during work for the workers [12]. The area related to nutritional status of workers has not been discussed.

It is assumed that the employers spend a quarter of their lives at work, and the pressure of work may affect their eating behaviour and lifestyle at work, which may lead to overweight and obesity, and in turn, overweight/obesity may affect their performance at work and may increase risk of different chronic diseases [13, 14]. In addition, different risk factors such as smoking, alcohol consumption, physical exercise, and other sociodemographic variables are the risk factors for overweight and obesity [15]. Some older studies conducted to assess the nutritional status among male adult workers in developing countries like India and Bangladesh showed that underweight was mostly prevalent and low overweight and obesity (less than 10%) were present [16, 17]. However, recent studies conducted in developing countries show that overweight and obesity among the industrial workers are in an increasing trend, i.e., more than 30% [18, 19].

In context of Nepal, the area for study related to assess the nutritional status of industrial workers is very new. Only research studies related to occupational diseases and the use of personal protective equipment have been conducted in case of Nepal. Hence, the objective of this study is to assess Factors Associated with the Nutritional Status among male workers of iron and steel industries in Bara District of Nepal.

## 2. Methods

### 2.1. Study Design, Study Population, and Sample Size

An industry-based quantitative descriptive cross-sectional study was done using a structured questionnaire cum interview schedule to assess the nutritional status and associated factors among male workers of iron and steel industries in Bara District. Bara District is one of the districts of Nepal where many industries are concentrated and consists of a higher number of iron and steel industries [20] that were chosen in this study. Informed written consent was obtained from all the participants who were above 19 years of age and agreed to participate in the study.

Sample size was calculated using

(1) $$\begin{array}{c} n=\frac{Z^{2}\cdot P\cdot Q}{L^{2}}, \end{array}$$

where *Z* is the value at 95% confidence level (i.e., 1.96∼2), *P* is the prevalence of obesity or overweight (17%) [6], *Q* is the probability of nonoccurrence of *p* (i.e., 1 − *P*), and *L* is the estimated error (5% or 0.05).

This gives the sample size of 271 with a nonresponse rate of 20%. There were a total of 11 iron and steel industries in Bara District [21]. Out of the total 11, four industries were randomly selected for the study. The selected industries with number of workers are given in Table 1.

For selecting total samples from each industry, the population proportionate sampling method was used. The samples were selected randomly in the industries by grouping the respondents into sedentary, moderate, and heavy workers by the population proportionate sampling method (Table 1). Out of the total samples, 57 sedentary workers, 180 moderate workers, and 34 heavy workers were selected by the population proportionate method.

### 2.2. Variables

**Figure 1:**
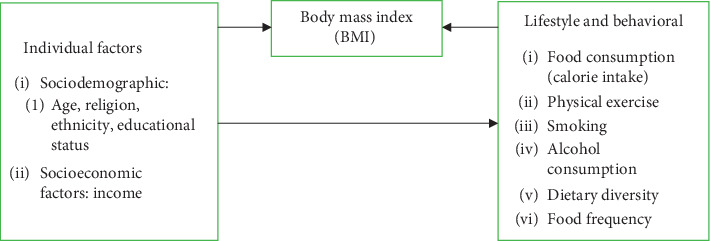
Conceptual framework of the study.

The nutritional status of the participants was determined based on the classification of the body mass index given by the WHO [22]. The dependant variable was categorized into BMI less than 25 kg/m^2^ as normal and BMI more than or equal to 25 kg/m^2^ as overweight/obese. Age of the industrial workers was categorised into less than 40 years and 40 years or more. Religion of the respondents was categorised into Hindu and others. Ethnicity was categorized into Brahmin/Chhetri, Madhesi, and Dalit/Janajati. Educational status was categorised into illiterate, primary level, secondary level, and higher than secondary level. Income has been categorised based on the salary scale of the Nepal Government. According to the government of Nepal, the least salary is NPR 19480 which is used to categorise the income of the workers [23]. Smoking, alcohol, and physical exercise were categorised into “Yes” and “No.” Dietary diversity was classified according to guidelines published by Kennedy et al. for measuring household and individual dietary diversity in Nutrition and Consumer Protection Division, the Food and Agriculture Organization of the United Nations in 2010, where foods are grouped into 3 categories such as lowest dietary diversity (≤3 food groups), medium dietary diversity (4 and 5 food groups), and high dietary diversity [24]. Sedentary work was measured by one questionnaire item (i.e., “how often does your job require you to sit for long periods of time during your work-shift?”) having a five-part Likert response set (all, most, some, little of the time, and never). For this analysis, the responses were grouped into high (all and most); middle (some); and low (little and never) [25].  Figure 1 shows the conceptual framework of the study.

### 2.3. Instrumentation and Data Collection

Stadiometer, Uniscale, and pretested structured questionnaire were tools, and anthropometric measurement, observation, interview and calculation were techniques used for the study. A face-to-face interview was conducted by the investigators themselves in local language of the respondents in the industries for assurance of data quality. The weight of the participants was measured in kilogram, and height was measured in the unit cm with nearest 0.1 cm. The 24-hour dietary recall method was used to calculate the total calories of the participants using a set of prestandardized vessels (cups).

### 2.4. Statistical Analysis

The data were recorded in Microsoft Excel sheet and were analyzed using Statistical Package for Social Sciences version 23. A descriptive analysis was done using mean, frequency, percentage, and standard deviation. Bivariate analysis was done using the chi-square test for categorical variables, and the *t*-test was used for numeric variables. Variables showing significance in bivariate analysis were included in the multivariate regression model. Significance level was observed at *P* value less than 0.05.

### 2.5. Ethical Consideration

Ethical consideration was obtained from the Research Committee of the College of Applied Food and Dairy Technology, Purbanchal University. Similarly, the consent was obtained from the administration section of the selected industries. The informed written consent from randomly selected individuals was also obtained during the assessment of nutritional status.

## 3. Results

Table 2 shows the sociodemographic characteristics of the participants. Among the total participants, more than half (55%) were below 40 years of age and more than nine-tenth (94.8%) of the participants followed Hindu religion. Almost half (46.9%) of the participants were Madhesi followed by Brahmin/Chhetri and Dalit/Janajati. More than three-fourth of the respondents were literate, and the proportion of participants with secondary level education was higher (39.1%). In Nepal, the lowest government salary scale is NPR. 19480. More than two-thirds (73.9%) of the participants had salary higher than the lowest government scale.

Table 3 shows the lifestyle nutrition-related behaviour of the participants. Almost 22% of the participants were recent smokers, and almost half (48%) used to drink alcohol. Only 22.5 percent of the participants used to do physical exercise. Regarding type of work, more than two-thirds (66.4%) of the participants were middle level sedentary workers. The food frequency of 62.4% participants was more than or equal to 4. More than half (56.8%) participants used to take 4–6 diverse food, and almost 20% used to take less diverse food.

**Figure 2:**
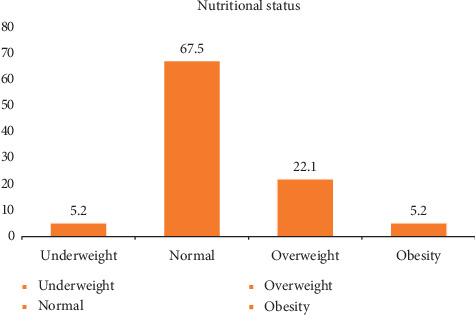
Nutritional status of the participants.

Figure 2 shows the nutritional status of the participants in which more than two-thirds (67.5%) were normal. More than three-fourths (27.3%) of the participants had BMI above normal.

Table 4 shows the bivariate and multivariate analysis of the nutritional status of the participants with different variables. Overweight/obesity was significantly associated with age of the participants in both bivariate and multivariate analyses. In multivariate, participants of age more than or equal to 40 were 2.54 (1.346–4.823) times more likely to be overweight/obese than the participants below 40 years of age. Religion was not significantly associated with nutritional status. Brahmins/Chhetri and Madhesi were more overweight/obese than the participants of Dalit/Janajati ethnicity. Brahmin/Chhetri were 6.14 (1.971–19.123), and Madhesi were 4.641 (1.534–14.04) times more overweight/obese than the Dalits/Janajati. No significant association was seen between nutritional status and education level of the participants. Participants with lower income were 1.5 times more overweight/obese than the participants with higher income, but it was statistically insignificant.

The participants who smoke were likely to be 4.165 (1.972–8.800) times more overweight/obese than the nonsmokers. Statistical significance was seen between alcohol and overweight/obesity in bivariate analysis, but it was near to significant in multivariate analysis. The participants who did not do physical exercise were 2.23 (1.068–4.680) times more overweight/obese, and it was significant with the nutritional status in the bivariate but was not significant in the multivariate analysis. The participants with low sedentary work were more likely to be overweight/obese than the middle and low sedentary workers, and it was statistically significant (*P* < 0.05). The participants having food frequency more than or equal to 4 was statistically significant with OR: 2.232 (1.101–4.522). Workers having more than 6 diverse food was significant with the nutritional status and were 3.679 (1.384–9.782) more overweight/obese than workers having 3 or less diverse food.

Table 5 shows the association between total calorie intake by the workers and nutritional status in which nutritional status of the workers was significantly associated with total calorie intake (*P* value < 0.001).

## 4. Discussion

The aim of the study was to find out the factors associated with the nutritional status of the male workers of iron and steel industries in Bara District of Nepal.

In this study, the prevalence of overweight and obesity was 22.1% and 5.2%, respectively. According to the Nepal Demographic and Health Survey 2016, the prevalence is lower than this study with 15% overweight and 3% obese [6]. The prevalence is lower as the survey was done among general people. The proportion of overweight is almost equal to a study carried out among government school teachers in Srinagar, India [26]. A study done among Kuwait Oil Company (KOC) employees revealed that the overall prevalence of overweight and obesity among KOC employees was 75% which is highly greater than the prevalence of this study [9]. The higher prevalence may be due to the nutritional habits of the workers and type of work in Kuwait oil company workers. A cross-sectional study conducted in Brazil among workers of different industries found that 19% of the male workers were obese and 42% were overweight which is almost double than our study [1]. Another study conducted in Kanpur India among workers working in the knitting industry revealed that 11% of the workers were overweight which is lower than our study and 10% were obese [27]. A study conducted among US workers showed that 27.7% of the workers met the BMI criteria of obesity which is higher than this study [28]. The dissimilar findings may be due to different lifestyle and nutritional habits of workers in the US as compared to Nepal. The obesity in general people in the US is higher (42.4%) than that of Nepal [29].

Age was significantly associated with the nutritional status in this study, and it is an evidence-based fact [30]. Similar type of result was shown by a study conducted in the US among different industrial workers [28]. Another study conducted among Japanese workers also revealed similar result [31]. Religion and ethnicity were also significant with nutritional status. The workers of upper ethnicity were more likely to be overweight/obese than the workers of lower ethnicity as the people of upper class have higher income than the Dalits/Janajatis. In a study conducted in the US reveled that non-Hispanic Asians were less likely to be obese than the workers with other ethnicity [28]. A survey conducted in India also supported the finding of this study [32].

Education was not associated with overweight/obesity of the workers in this study and is supported by other studies [33, 34]. The illiterate workers may not have knowledge related to risk factors of overweight and obesity. In this study, the illiterate people had high alcohol consumption and low physical exercise than the literate. A study conducted among US workers showed that education was significant with the nutritional status of the workers [28]. Income was not significant with the nutritional status and is in agreement with other studies [33, 35]. The workers with low income also used to consume higher calories than the workers with high income, resulting in high prevalence of overweight and obesity. In a developing country like Nepal due to urbanization, diets rich in fibre and complex carbohydrates are being replaced by the diets rich in sugars and fats, resulting in increased overweight and obesity [36]. Data also have shown that in cost constraint situation, people prefer food higher in the proportion of energy derived from cereals, sweets, and added fats rather than form vegetables and fruits [37].

Smoking was significantly related to nutritional status of factory workers with *P* value less than 0.001. The study is supported by a cross-sectional study carried out in the UK among general people, which showed that current heavy smokers (>20 cigarettes per day) were more likely to be obese than both moderate (10–20 cigarettes per day) and light (<10 cigarettes per day) smokers, and moderate smokers were more likely to be obese than light smokers [38]. A meta-analysis done by Dallongevilli et al. found that smokers declared significantly higher intakes of energy, total fat, saturated fat, cholesterol, and alcohol than nonsmokers [39]. Consumption of alcohol was significant in bivariate analysis and is supported by a Nigerian study [33]. Consumption of alcohol was not significant in multivariate analysis. Other studies carried also supported the finding [31, 40]. Another study conducted among Spanish graduates was not in agreement with this study which may be differences between the type of people and the type of work [34]. Physical exercise was significantly associated with prevalence of overweight/obesity in bivariate analysis. The study is supported by the finding of a cross-sectional study conducted in Ghana and Nigeria [33, 41]. The result was not in agreement with the same study conducted in Ghana in multivariate analysis [41]. The workers with age less than 40 years are more likely to do physical exercise than the older workers in the study.

In this study, sedentary work was significant with nutritional status and is in agreement with a study conducted in the US in which long sitting time was significantly associated with BMI of the participants [42] but was not significant in a study conducted in Ghana [41]. Another study conducted among US workers supported the finding [25]. According to the WHO, higher the sedentary work, more the risk of being overweight or obese in people with sedentary lifestyle [43]. Food frequency was significantly associated with overweight/obesity and is in agreement with the study conducted in Kerala among geriatric population [44]. A survey conducted in the US which used the data from the USDA Continuing Survey of Food Intake by Individuals (CSFII) collected in 1994–1996 supported the finding in which eating frequency of more than three times was associated with overweight or obese [45].

Dietary diversity was significantly associated with nutritional status of the workers, and the study is supported by a study carried out in Southwest China [46]. There was disagreement with another study conducted among nurses in Iran [47] which may be due to the gender differences and type of work and types of food taken. Another study conducted in Nigeria concluded that the overweight and obesity were higher in the adults with medium dietary score, but it was not significant [48]. Other studies conducted among adults of Sri Lanka and Brazil supported the findings in which excess weight gain is associated with higher dietary diverse foods [49, 50]. In this study, the total mean calorie intake among the overweight and obese is higher which may be due to the higher diverse food. The higher diverse food may contain higher calories, resulting in the increased BMI. Total calorie intake was significantly associated with nutritional status and is supported by a study conducted in office workers of Korea [51].

The study has some limitations: it could not include female workers as there were no females working in the iron and steel industries. The risk factors related to overweight and obesity were assessed on the basis of self-reported data by the industrial workers only, without experimental measurements.

## 5. Conclusion

The study has presented high prevalence of overweight/obesity among workers in iron and steel industries of Bara District. More importantly, findings of the study have pinpointed towards socioeconomic, sociodemographic, lifestyle, and dietary factors that may increase the risk of overweight or obesity in workers. Many of the workers lacked physical exercise and were moderate to sedentary workers. The workers who had more diverse food had increased overweight and obesity, and the higher diverse food may contain higher calories and, in absence of physical exercise, may have resulted in increased overweight and obesity.

The findings of the study show that there is need for proper nutrition education to the industrial workers which may contribute to change in the lifestyle and behaviours of the workers and should be more focused among the workers of age more than 40 years and with lower socioeconomic class. There is need for further research studies in the field of nutrition in industries in case of developing countries such as Nepal.

## Figures and Tables

**Table 1:** Sample size of the study.

Industry no.	Total number of workers				Samples selected			
	Sedentary	Moderate	Heavy	Total	Sedentary	Moderate	Heavy	Total
1	179	565	106	850	33	104	20	157
2	97	306	57	460	18	56	11	85
3	19	60	11	90	3	11	2	16
4	15	46	9	70	3	9	1	13
Total	310	977	183	1470	57	180	34	271

**Table 2:** Sociodemographic and socioeconomic characteristics of the participants (*N* = 271).

Characteristics	Frequency	Percentage
Age (median = 37, SD = 8.2)		
Less than 40	149	55
More than or equal to 40	122	45

Religion		
Hindu	257	94.8
Others	14	4.2

Ethnicity		
Brahmin/Chhetri	97	35.8
Madhesi	127	46.9
Dalit/Janajati	47	17.3

Educational status		
Illiterate	57	21
Primary	82	30.3
Secondary	106	39.1
Higher than secondary	26	9.6

Income (median = NPR. 25000, SD = 16110.45)		
Less than NPR. 19480	73	26.9
More than NPR. 19480	198	73.9

**Table 3:** Lifestyle and nutritional behavioural characteristics (*N* = 271).

Characteristics	Frequency	Percentage
Smoking		
Yes	59	21.8
No	212	78.2
Alcohol		
Yes	130	48
No	141	52
Physical exercise		
Yes	61	22.5
No	210	77.5
Sedentary work		
High	57	21.0
Middle	180	66.4
Low	34	12.5
Food frequency		
Less than 4	102	37.6
More than or equal to 4	169	62.4
Dietary diversity		
Less than 4	53	19.6
4–6	154	56.8
More than 6	64	23.6
Body mass index: (mean = 23.72, SD = 3.47)		
Energy intake: (mean = 2167.7, SD = 356.56)		

**Table 4:** Bivariate and Multivariate logistic analysis of the nutritional status of the participants.

Characteristics	Nutritional status		COR (95% CI)	*P* value	AOR (95% CI)	*P* value
	Normal	Overweight/obesity				
Age						
Less than 40	120	28	1		1	
More than or equal to 40	77	46	2.56 (1.47–4.43)	0.001^*∗*^	2.548 (1.346–4.823)	0.004^*∗*^

Religion						
Hindu	188	69	1			
Others	9	5	1.51 (0.49–4.67)	0.471		

Ethnicity						
Brahmin/Chhetri	65	32	3.36 (1.294–8.747)	0.013^*∗*^	6.14 (1.971–19.123)	0.002^*∗*^
Madhesi	91	36	2.703 (1.056–6.918)	0.038^*∗*^	4.641 (1.534–14.04)	0.007^*∗*^
Dalit/Janajati	41	6	1		1	

Educational status						
Illiterate	37	20	1			
Primary	65	17	0.484 (0.226–1.037)	0.062		
Secondary	78	28	0.664 (0.332–1.330)	0.248		
Higher than secondary	17	9	0.979 (0.37–2.59)	0.967		

Income						
Less than NPR. 19480	48	25	1.584 (0.886–2.832)	0.121		
More than NPR. 19480	149	49	1			

Smoking						
Yes	32	27	2.92 (1.616–5.43)	<0.001^*∗*^	4.165 (1.972–8.800)	<0.001^*∗*^
No	165	47	1		1	

Alcohol						
Yes	85	45	2.045 (1.185–3.52)	0.010^*∗*^	1.841 (0.975–3.479)	0.060
No	112	29	1		1	

Physical exercise						
Yes	51	10	1		1	
No	146	64	2.236 (1.068–4.680)	0.030^*∗*^	1.897 (0.787–4.569)	0.154

Sedentary work						
High	31	26	1		1	
Middle	142	38	0.319 (0.170–0.60)	<0.001^*∗*^	0.269 (0.126–0.575)	0.001^*∗*^
Low	24	10	0.497 (0.201–1.226)	0.129	0.246 (0.083–0.732)	0.012^*∗*^

Food frequency						
Less than 4	84	18	1		1	
More than or equal to 4	113	56	2.313 (1.267–4.220)	0.006^*∗*^	2.232 (1.101–4.522)	0.026^*∗*^

Dietary diversity						
Less than 4	42	11	1		1	
4–6	120	34	1.082 (0.503–2.326)	0.840	0.955 (0.390–2.337)	0.919
More than 6	35	29	3.164 (1.385–7.229)	0.006^*∗*^	3.679 (1.384–9.782)	0.009^*∗*^

COR, crude odds ratio; AOR, adjusted odds ratio; CI, confidence interval; ^*∗*^*P* value <0.05.

**Table 5:** Independent *t*-test of nutritional status with total energy intake (total calories per day).

Characteristics	Mean	SD	CI	*P* value
Normal	2085.56	312.037	−389.756 to −212.100	<0.001^*∗*^
Overweight/obesity	2386.49	376.929		

CI, confidence interval, ^*∗*^*P* < 0.05.

## Data Availability

The data used to support the findings of this study are included within the supplementary information file.

## Supplementary Materials

Supplementary Materials The supplementary file contains the SPSS data used in the study.
